# Incidence and Survival of urothelial carcinoma of the urinary bladder in Norway 1981-2014

**DOI:** 10.1186/s12885-016-2832-x

**Published:** 2016-10-13

**Authors:** B. K. Andreassen, B. Aagnes, R. Gislefoss, M. Andreassen, R. Wahlqvist

**Affiliations:** 1 Department of Research, Cancer Registry of Norway, Institute for Population-based Research, Oslo, Norway; 2 Department of Pathology, Vestre Viken Hospital Trust, Drammen, Norway; 3 Department of Urology, Oslo University Hospital, Oslo, Norway; 4 Department of Registration, Cancer Registry of Norway, Institute for Population-based Research, Oslo, Norway

**Keywords:** Urothelial carcinoma of the urinary bladder, Bladder cancer, Incidence, Relative survival, Trends, Registry data, Epidemiology

## Abstract

**Background:**

Urothelial carcinoma of the urinary bladder (UCB) is the 4^th^ most common cancer type in men in developed countries, and tumor recurrence or progression occurs in more than half of the patients. Previous studies report contradictory trends in incidence and survival over the past decades. This article describes the trends of UCB incidence and survival from 1981 to 2014, including both invasive and non-invasive UCB using data from the Cancer Registry of Norway.

**Methods:**

In Norway, 33,761 patients were diagnosed with UCB between 1981 and 2014. Incidence and 5-year relative survival were calculated, stratified by sex, morphology, stage, age and diagnostic period. Age-period-cohort models were used to distinguish period- and cohort effects. Temporal trends were summarized by calculating the average absolute annual change in incidence and relative survival allowing for breaks in this trend by incorporating a joinpoint analysis. Excess mortality rate ratios (EMRR) quantify the relative risks by using a proportional excess hazard model.

**Results:**

The incidence of UCB in men increased from 18.5 (1981-85) to 21.1 (1991-95) per 100 000 person-years and was rather stable thereafter (1996–2014). The incidence rates of UCB were lower in women increasing linearly from 4.7 to 6.2 over the past 34 years (*p* = 5.9 · 10^-7^). These trends could be explained by an increase of the incidence rates of non-invasive tumors. Furthermore, the observed pattern seemed to represent a birth cohort effect. Five-year relative survival increased annually with 0.004 in men (*p* = 1.3 · 10^-6^) and 0.003 in women (*p* = 4.5 · 10^-6^). There is a significant increase over the past 34 years in survival of UCB in both genders for local tumors but not for advanced stages.

**Conclusions:**

Increasing and stable incidence trends mirror little improvement in primary and secondary prevention of UCB for more than three decades. Survival proportions increased only marginally. Thus, any changes in treatment and follow-up care did not lead to notable improvement with respect to survival of the patients. High estimates of preventable cases together with large recurrence rates of this particular cancer type, demand more research on prevention guidelines, diagnostic tools and treatment for UCB.

## Background

Despite large global differences in the incidence of urothelial carcinoma of the urinary bladder (UCB), this cancer type remains the second most common genitourinary malignancy after prostate cancer in men worldwide. Globally, about 330 000 new UCB cases were diagnosed and 123 000 UCB patients died from the disease in 2012. UCB is most frequent in Europe, Northern America, Western Asia and Northern Africa and least common in Eastern, Western and Middle Africa, Central America and the non-western regions of Asia [1]. Within Europe, UCB is the fourth most common cancer type in men with an age-standardized incidence rate (world) of 17.7 per 100 000 person-years [1]. The corresponding rate in Norway is 20.8 per 100 000 person-years based on the diagnosis period from 2009 to 2013 [2]. UCB incidence in women is much lower with 3.5 cases per 100 000 person-years in Europe and 6.4 in Norway. International variation in UCB incidence have recently been described in detail [3], also including trends over time.

Several risk factors have been identified for UCB, tobacco smoking being the most predominant one. The population attributable risk for ever smoking has lately been estimated to be approximately 50 % in both men and women in the US [4] and nearly 40 % in the UK [5]. Men are more likely to get UCB with a male to female ratio of 3.2 in Norway [2]. UCB is also related to occupational exposure to certain chemicals like aromatic amines, chlorinated hydrocarbons and polycyclic aromatic hydrocarbons [6, 7]. Patients previously irradiated for pelvic and abdominal malignancies are also at increased risk, as is also shown for intake of certain drugs used in previous cancer treatment [8] as well as diabetes medication [9]. UCB rarely occurs before the age of 40, and has a median age of diagnosis at 75 in the UK [10].

In Norway, 95 % of all UCB cases are of the transitional cell type, and about 60 % are primarily diagnosed without invasion into subepithelial connective tissue (tumor stage T1) or muscle (T2-4). These non-invasive tumors (papillary: Ta, Carcinoma in situ: Tis) and dysplasia are characterized by its high recurrence rates after transurethral resection of an initial tumor. Non-invasive papillary (Ta) tumors form the largest group and account for half of all diagnosed urothelial carcinomas. The majority of these tumors will recur, but the risk of progression to invasive UCB is low (4–7 %) for low-grade Ta tumors and approximately 12–23 % for high-grade Ta tumors [11, 12]. Carcinoma in situ seems to be more likely to progress than non-invasive papillary tumors, especially if concurrent with papillary tumors [12, 13]. The high recurrence rates and the low but imminent progression risk leads to a tight follow-up of the patients with frequent visits and resource demanding treatment as well. This makes UCB one of the most expensive cancers to treat on a per-patient basis [14] in addition to being bothersome for the patients.

Incidence and survival trends for UCB in different countries based on cancer registry data have been described previously [15–17]. However, inclusion and exclusion criteria differ with respect to diagnose groups and periods as well as classification and registration practices. Furthermore, to our knowledge, none of the articles addressing UCB trends used a nationwide registry. We will describe the trends of UCB incidence and survival over time in Norway for all patients diagnosed with urothelial carcinoma of the urinary bladder between 1981 and 2014, including both invasive and non-invasive UCB (including dysplasia) using data from the Cancer Registry of Norway.

## Methods

### Material

The Cancer Registry of Norway has since 1953, compulsory by law, registered virtually all new cancer diagnoses in Norway. The registry receives information from three independent sources (clinicians, pathology laboratories, and from the Cause of Death Registry), which ensures completeness and high quality data [18]. Patients are identified through the unique national personal identification number assigned to all newborns and residents in Norway since 1960. The present study comprises all new cases of histologically verified invasive and non-invasive urothelial carcinoma of the urinary bladder in the Norwegian population diagnosed between 1981 and 2014. UCB cases were selected based on morphological codes for the transitional cell type as presented in Table 1. UCB patients diagnosed before 1981 were excluded due to registration changes in the seventies mainly for non-invasive tumors. Thus, in total, 33,761 UCB patients were included in this study (Table 1). Participants were followed until death, migration or end of follow up on the 31^st^ of March 2016. The total follow-up time was 230 783 person-years with a median follow-up time of 15.0 years. Out of all 33,761 UCB patients included in this study, 5 228 individuals died from UCB according to the cause of death certificate.

**Table 1:** Patient inclusion criteria

Morphology code	Description	Morphology group	Number of individuals	Percentage
8130	Papillary, mild dysplasia, non-invasive (WHO-grade I)	Non-inv pap carcinoma LG	6752	20.0 %
8131	Papillary, moderate dysplasia, non-invasive (WHO-grade II)	Non-inv pap carcinoma LG	7644	22.6 %
8136	Papillary, not otherwise specified (NOS), non-invasive	Non-inv pap carcinoma LG	238	0.7 %
8132	Papillary, non-invasive, high-grade (WHO-grade III)	Non-inv pap carcinoma HG	2400	7.1 %
8120	Non-papillary, mild dysplasia, non-invasive (WHO-grade I)	Dysplasia	303	0.9 %
8121	Non-papillary, moderate dysplasia, non-invasive (WHO-grade II)	Dysplasia	589	1.7 %
8126	Non-papillary, NOS, non-invasive	Dysplasia	441	1.3 %
8122	Non-papillary, non-invasive, high-grade (WHO-grade III)	Carcinoma in situ	1198	3.5 %
8123	Non-papillary, NOS, invasive	Inv carcinoma	319	0.9 %
8124	Non-papillary, mild dysplasia, invasive (WHO-grade I)	Inv carcinoma	28	0.1 %
8125	Non-papillary,moderate dysplasia, invasive (WHO-grade II)	Inv carcinoma	758	2.2 %
8127	Non-papillary, invasive, high-grade (WHO-grade III)	Inv carcinoma	3471	10.3 %
8133	Papillary, NOS, invasive	Inv carcinoma	169	0.5 %
8134	Papillary, mild dysplasia, invasive (WHO-grade I)	Inv carcinoma	237	0.7 %
8135	Papillary, moderate dysplasia, invasive (WHO-grade II)	Inv carcinoma	2205	6.5 %
8137	Papillary, invasive, high-grade (WHO-grade III)	Inv carcinoma	3304	9.8 %
80103^a^	Carcinoma in situ, NOS	Inv carcinoma	185	0.5 %
80203^a^	Carcinoma, undifferentiated, invasive, NOS	Inv carcinoma	91	0.3 %
812031^a^	Highly differentiated, invasive, G1, low-grade	Inv carcinoma	84	0.2 %
812032^a^	Moderately differentiated, invasive, G2, low-grade	Inv carcinoma	193	0.6 %
812033^a^	Poorly differentiated, invasive, G3, high-grade	Inv carcinoma	2180	6.5 %
812034^a^	Undifferentiated, invasive, G4, high-grade	Inv carcinoma	27	0.1 %
812039^a^	NOS, invasive	Inv carcinoma	945	2.8 %
		Total number of individuals	33761	

Morphology codes (MOTNAC: Manual of Tumor Nomenclature and Coding from 1951 from the American Cancer Society), description, morphology groups and corresponding number and percentage of individuals included in this study

^a^ICD-O: International Classification of Diseases for Oncology 1976-

Information on morphology, stage, grade, sex, age at diagnosis and date of diagnosis were retrieved from the Cancer Registry of Norway. Morphology, stage and grade were defined based on the most severe diagnosis within a 5-month window including the first of the month when the first UCB diagnosis was received. We define five morphology groups based on the available tumor categories and grade information: Non-invasive papillary carcinoma low- and high-grade (Ta), non-invasive flat carcinoma (Tis), dysplasia (low-grade flat carcinoma) and invasive carcinoma (T1-T4). Grade information is based on WHO 1973 [19] and grouped into low (LG, WHO grade 1 and 2)- and high-grade (HG, WHO grade 3) (see Table 1). Stage is categorizsed as localized (non-invasive/invasive cancer without any metastases), regional advanced (any infiltration into surrounding areas or regional metastases) and distant advanced (distant metastases) tumors. For the presentation of the results, age at diagnosis was divided into four age groups (≤49, 50–64, 65–79, ≥80). The year of diagnosis is grouped into 5-years intervals (diagnostic periods): 1981–1985, 1986–1990, …, 2006–2010 and 2011–2014.

### Statistics

Incidence rates (per 100 000 person-years) were calculated based on the number of individuals getting their first UCB diagnosis and the number of individuals living in Norway for a certain sex, diagnostic time-period and age group. Age-specific incidence rates are presented for each of the four age-groups stratified for sex. Direct age-standardized incidence rates were calculated applying the World Standard Population [20] according to sex as well as age group, morphology group and stage across all diagnostic periods. Temporal trends of the incidence rates were best represented by a linear model, where the estimated regression coefficient ${\widehat{\beta}}_I$ represents the average absolute annual incidence change. In order to summarize the observed trend over the last 34 years, this parameter has been provided together with the standard error and *p*-values for the test of an incidence change over time. We also implemented a joinpoint analysis [21] to uncover trends, which change over time. In order to interpret trends in age-specific incidence rates over time, we also apply an extension of the age-period-cohort model [22] to separate diagnosis period and birth cohort effects. An APC model incorporating restricted cubic splines, implemented in Stata was used [23, 24]. The APC model was applied for male patients with an age at diagnosis between 70 and 80, diagnosed between 1981 and 2010 and born between 1910 and 1930, which means that the model includes reliable information from the birth cohorts of interest.

5-year relative survival, based on the cohort approach, was estimated using the age-standardardized Ederer II method applying national population life-tables by sex, age group and diagnosis period [25]. The internal age-standardization used the age distribution of the last diagnostic period 2011-14 as weights. Temporal trends of 5-year relative survival rates were best represented by a linear model. Thus, the average absolute change in annual 5-year relative survival estimates was provided together with the standard error and *p*-values for the test of a significant trend. We also implemented a joinpoint analysis [21] to uncover those trends, which change over time. The annual change in 5-year survival proportions was estimated by the regression coefficient ${\widehat{\beta}}_{RS}.$ Because follow-up data for the latest diagnostic period are lacking, 5-year survival estimates for 2011-14 are based on a period approach. The corresponding column is marked with an (*) in order to emphasize the differently derived estimates and these estimates have not been included in the trend analysis.

We fitted a proportional excess hazard model [26–28] where sex, diagnostic period, age group, morphology group and stage were included as categorical variables. The baseline hazard was modelled using 5df for the spline variables using the Stata command stpm2 [29]. Excess mortality rates estimate the absolute difference between the expected mortality rate (from lifetables) and the observed mortality rate (from the data). The ratio of these quantities, the excess mortality rate ratios (EMRR), are in their interpretation similar to the hazard rate ratio and thus represent the factor of which patients under a certain condition are more likely to die compared to patients under another condition. EMRRs are reported together with 95 % confidence intervals (CI).

All statistical analyses were performed in Stata 14/MP for Windows [30].

## Results

### Overview

A total number of 33,761 cases was diagnosed with UCB (transitional cell type) between 1981 and 2014. An overview over these patients with respect to sex as well as vital status (UCB and other cancer related death, alive, unknown), morphology group, stage, and age by sex and combined is provided in Table 2. Three quarters of the UCB patients diagnosed between 1981 and 2014 were men. For all patients (men and women combined), the median age at diagnosis was 72 years (inter-quartile range: 64–79 years). The majority of patients (50.5 %) were diagnosed with non-invasive papillary carcinoma Ta (43.4 % low-grade, 7.1 % high-grade). Invasive transitional carcinoma (T1-T4) accounted for 42.0 % of all UCB cases in this study and carcinoma in situ (3.6 %) as well as dysplasia (3.9 %) are the least frequent morphology groups. Out of all patients diagnosed between 1981 and 2014, 66.6 % had died by 31^st^ of March 2016, either due to UCB (15.5 %), cancer (not UCB) (22.0 %) or causes other than cancer (29.1 %). Women had relatively more invasive (44.3 vs 41.3 %) and more advanced (10.0 vs 8.5 %) tumors than men.

**Table 2:** Patient characteristics

	Men	Women	All
%	75.6	24.4	100.0
Age [25–75 % percentile]	72 [64–79]	73 [65–81]	72 [64–79]
Morphology			
Non-inv pap trans carcinoma low-grade	43.4	43.0	43.4
Non-inv pap trans carcinoma high-grade	7.6	5.6	7.1
Displasia	3.9	4.1	3.9
Carcinoma in situ	3.7	3.1	3.6
Inv trans carcinoma	41.3	44.3	42.0
Stage			
Localised	91.5	89.9	91.1
Regional advanced	5.6	5.9	5.7
Distant advanced	2.9	4.1	3.2
Death			
Bladder cancer	15.0	17.2	15.5
Other cancer	22.1	21.4	22.0
Other than cancer	30.2	26.2	29.1
Unknown	2.7	2.6	2.8
Alive	30.0	32.6	30.6

Distribution of age, morphology group, stage and cause of death by sex and in total in the study population of patients diagnosed with urothelial carcinoma of the urinary bladder in Norway from 1981 to 2014

### Incidence

Age-standardized incidence rates (World) are summarized in seven diagnostic periods in Table 3. In addition, estimates for the average annual incidence change $\left({\widehat{\beta}}_I\right)$ are provided. These quantities estimate the average incidence trend throughout the study period and give an indicator for whether (and how much) incidence changed linearly over the past 34 years. For men, after a slightly increasing trend in the 1980s, UCB incidence has been stable throughout the remaining study period. A joinpoint analysis revealed an increasing trend of 0.34 (*p* = 0.048) from 1981-89 and a stable trend from 1990 to 2014 (${\widehat{\beta}}_I$ =0.02, *p* = 0.531). Thus, on average, the incidence increased by 0.34 $\left({\widehat{\beta}}_I\right)$ within a year or 2.7 from 1981 to 1989. The remaining 26 years of the study period, the incidence increased on average 0.02 per year or 0.5 from 1990 to 2014. The incidence of UCB in men was between 18.3 and 21.4 per 100 000 person-years for all diagnostic periods during the study period. The incidence of UCB was lower in women with incidence rates increasing from 4.7 to 6.2 over the past 34 years. The corresponding trend was significant: ${\widehat{\beta}}_I$ = 0.043 (*p* = 5.9 · 10^-7^).

**Table 3:** Incidence rates

		1981-1985	1986-1990	1991-1995	1996-2000	2001-2005	2006-2010	2011-2014	*β*_*I*_ *(SE)*	*p*-value
Men	*n*	2972	3321	3628	3575	3928	4192	3915		
		18.5	19.7	21.1	19.7	20.6	20.4	21.4	0.070(0.021)	0.002^a^
Women	*n*	974	1038	1110	1150	1262	1380	1316		
		4.7	4.9	5.2	5.1	5.3	5.7	6.2	0.043(0.007)	5.9E-07^a^
Men	Age (years)									
	0–49	2.0	1.8	2.1	2.0	1.9	1.8	2.5	0.008(0.007)	0.246
	50–64	46.2	49.0	50.4	40.3	42.4	42.6	44.4	−0.196(0.102)	0.065
	65–79	148.4	156.0	171.0	169.1	175.4	176.1	173.1	0.899(0.186)	3.2E-05^a^
	80+	194.1	256.2	263.1	268.0	301.8	286.1	317.8	3.47(0.45)	7.1E-09^a^
	Morphology									
	Non-inv pap carcinoma LG	8.7	9.5	9.9	9.2	9.5	8.9	8.3	−0.016(0.016)	0.335
	Non-inv pap carcinoma HG	0.6	0.9	1.3	0.9	1.4	2.3	2.6	0.061(0.006)	1.5E-10^a^
	Dysplasia	0.6	0.7	1.0	0.9	0.6	0.6	1.4	0.011(0.006)	0.070
	Carcinoma in situ	0.4	0.7	0.7	0.9	0.9	0.9	0.7	0.013(0.003)	0.002^a^
	Inv carcinoma	8.2	8.0	8.2	7.8	8.2	7.7	8.4	−0.001(0.010)	0.985
	Stage									
	Localised	16.6	17.9	19.6	18.2	18.6	18.5	19.9	0.072(0.021)	0.001^a^
	Regional advanced	1.3	1.1	1.0	0.8	1.3	1.4	1.0	0.001(0.005)	0.919
	Distant advanced	0.6	0.7	0.5	0.7	0.7	0.6	0.4	−0.003(0.003)	0.380
Women	Age (years)									
	0–49	0.7	0.7	0.8	0.7	0.6	0.7	0.8	0.001(0.004)	0.739
	50–64	12.3	13.0	12.8	11.6	13.8	14.1	15.0	0.083(0.034)	0.021^a^
	65–79	34.2	35.7	39.2	41.3	41.2	44.8	47.5	0.425(0.061)	6.4E-08^a^
	80+	57.0	52.9	50.8	52.3	61.2	61.4	76.3	0.519(0.167)	0.004^a^
	Morphology									
	Non-inv pap carcinoma LG	2.1	2.4	2.4	2.5	2.8	2.8	2.6	0.022(0.005)	1.0E-04^a^
	Non-inv pap carcinoma HG	0.1	0.1	0.3	0.2	0.2	0.4	0.6	0.014(0.002)	5.4E-08^a^
	Dysplasia	0.2	0.2	0.2	0.2	0.2	0.2	0.6	0.006(0.003)	0.037^a^
	Carcinoma in situ	0.1	0.2	0.1	0.2	0.2	0.2	0.1	0.002(0.001)	0.233
	Inv carcinoma	2.3	2.0	2.2	1.9	2.0	2.1	2.3	−0.001(0.005)	0.823
	Stage									
	Localised	4.2	4.4	4.7	4.5	4.7	5.0	5.7	0.040(0.008)	8.6E-06^a^
	Regional advanced	0.4	0.3	0.2	0.3	0.4	0.4	0.3	0.001(0.001)	0.611
	Distant advanced	0.2	0.2	0.2	0.2	0.2	0.3	0.2	0.002(0.001)	0.172

Age-standardized incidence rates (applying the World Standard Population) by sex, morphology group and stage as well as age-specific incidence rates stratified for 5-years interval of diagnosis from 1981 to 2014. Average annual incidence changes $\left({\widehat{\beta}}_I\right)$ are reported together with standard error (SE) and the *p*-value for the test of a significant incidence trend. Significant *p*-values (based on the 5 % threshold) are marked with^a^

**Fig. 1:**
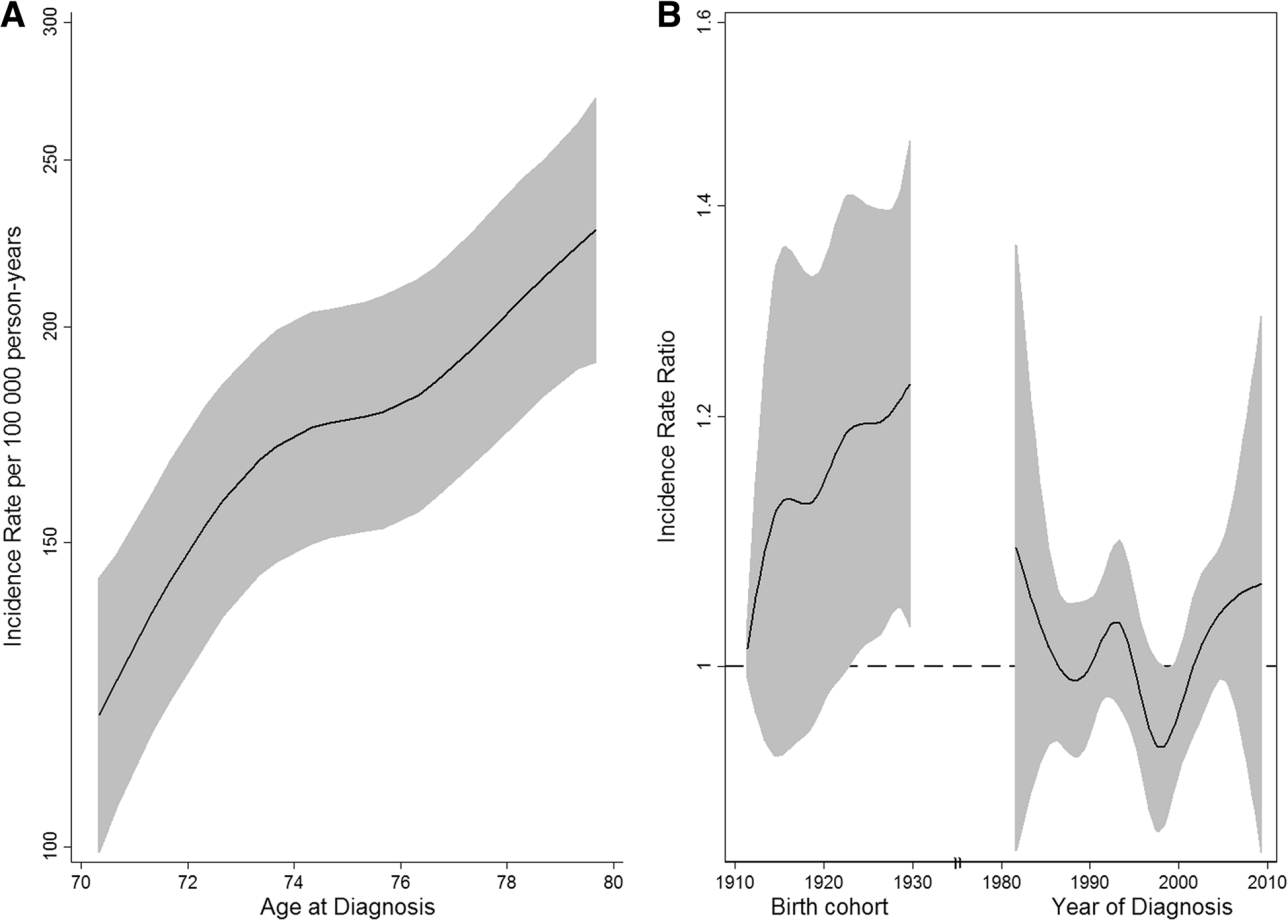
Age-standardized incidence rates per 100 000 person-years (**a**) and incidence rate ratios for birth cohort and diagnostic period effects for men diagnosed with urothelial carcinoma of the urinary bladder in Norway (**b**)

In men, age-specific incidence rates were increasing during the past 34 years, especially for the oldest patients (${\widehat{\beta}}_I$ = 3.5, *p* = 7.1 · 10^-9^) and those between 65 and 79 (${\widehat{\beta}}_I$ = 0.9, *p* = 3.2 · 10^-5^). Similar age-dependent incidence trends could also be seen in women, although less pronounced probably due to the lower number of cases. This observed pattern was most likely mainly due to a birth cohort effect as illustrated in Fig. 1. The APC model confirmed the age-effect (Fig. 1a) and suggested a larger birth cohort effect compared to the diagnosis period effect (Fig. 1b). The incidence rate ratio (IRR) was 1.22 (CI: 1.04–1.45) when comparing the 1930 birth cohort to the one from 1910. This particular birth cohort effect stayed the same when reducing the model to an age-cohort model (results not shown).

In men, there was a significant increase in high-grade non-invasive papillary carcinoma (Ta HG: *p* = 1.5 · 10^-10^) over the study period. Age-standardized incidence rates in men have been rather stable over the whole observation period for invasive carcinoma (*p* = 0.985) and low-grade non-invasive papillary carcinoma (Ta LG *p* = 0.335). The incidence of both high- and low-grade non-invasive papillary carcinoma (Ta) was increasing in women (HG *p* = 5.4 · 10^-8^, LG *p* = 1.0 · 10^-4^). The corresponding increase of incidence rates for carcinoma in situ has been less pronounced in women (*p* = 0.233), possibly due to small numbers of cases. A joinpoint analysis reveals that the increase in incidence rates of high-grade non-invasive papillary carcinoma is rather small until 2001 (men: *p* = 0.055) and 2003 (women: *p* = 0.105), but significant thereafter (men: *p* = 1.0 · 10^-8^, women: men: *p* = 1.0 · 10^-8^).

Both genders showed an increasing trend with respect to the incidence rates for localized cancers. This trend was more pronounced for women (*p* = 8.6 · 10^-6^) than men (*p* = 0.001). Cases with advanced stage were rather rare, such that the interpretation suffered from small number of cases in both men and women. Still, there is a decreasing tendency for distant advanced tumors in men (*p* = 0.380), while the corresponding tendency in women is increasing (*p* = 0.172).

### Relative Survival

Table 4 presents the sex-specific 5-year relative survival across 5-year diagnosis intervals from 1981 to 2014 stratified for age and morphology group as well as stage. Furthermore, the average annual survival change $\left({\widehat{\beta}}_{RS}\right)$ estimates the average trend of the 5-year relative survival proportions throughout the study period. It gives an indicator for whether (and how much) survival changed over the past 30 years. On average, 5-year relative survival proportions increased annually by ${\widehat{\beta}}_{RS}$ =0.004 in men (*p* = 1.3 · 10^-6^) and ${\widehat{\beta}}_{RS}$ =0.003 in women (*p* = 4.5 · 10^-6^). In men, the 5-year relative survival was 0.67 (CI: 0.64–0.70) in 1981-85 and 0.77 (CI: 0.75–0.79) in 2006-10. The corresponding 5-year relative survival in women were 0.63 (CI: 0.59–0.67) for 1981-85 and 0.72 (CI: 0.69–0.75) in 2006-10. The age-specific 5-year relative survival in men stayed mainly stable within the youngest age group (≤50 years), while improvement in relative survival was the more pronounced the older the patient was (≥80 years: ${\widehat{\beta}}_{RS}$ =0.007, *p* = 0.015). In women, these trends were similar except that the increase in 5-year relative survival was most distinct in the age group 65–79 years (${\widehat{\beta}}_{RS}$ =0.008, *p* = 1.5 · 10^-5^). There was a significant increase in 5-year relative survival for invasive carcinomas in both men (${\widehat{\beta}}_{RS}$ =0.003, *p* = 2.3 · 10^-4^) and women (${\widehat{\beta}}_{RS}$ =0.003, *p* = 0.010). Relative survival for localized stages were significantly larger towards the end of the study period in both men (${\widehat{\beta}}_{RS}$ =0.004, *p* = 7.0 · 10^-6^) and women (${\widehat{\beta}}_{RS}$ =0.004, *p* = 9.2 · 10^-6^). There is no significant increase in survival for advanced stages, the tendency is towards decreasing survival for the distant advanced stage in both genders. Joinpoint analysis did not reveal any significant changes in the trend over time.

**Table 4:** Relative survival proportions

		1981-85		1986-90		1991-95		1996-2000		2001-05		2006-10		2011-14^a^		*β*_*RS*_ *(SE)*	*p*-value
	Men	0.67	(0.64,0.70)	0.71	(0.68,0.74)	0.73	(0.70,0.76)	0.72	(0.70,0.75)	0.75	(0.72,0.77)	0.77	(0.75,0.79)	0.77	(0.75,0.79)	0.004 (0.001)	1.3E-06^b^
	Women	0.63	(0.59,0.67)	0.68	(0.64,0.72)	0.65	(0.61,0.69)	0.70	(0.67,0.74)	0.70	(0.67,0.73)	0.72	(0.69,0.75)	0.74	(0.71,0.77)	0.003 (0.001)	4.5E-06^b^
Men	Age (years)																
	0–49	0.90	(0.84,0.95)	0.85	(0.78,0.90)	0.93	(0.88,0.97)	0.92	(0.86,0.95)	0.89	(0.83,0.93)	0.91	(0.85,0.95)	0.94	(0.84,0.98)	0.001 (0.001)	0.623
	50–64	0.78	(0.74,0.81)	0.81	(0.78,0.85)	0.83	(0.80,0.86)	0.84	(0.80,0.87)	0.84	(0.81,0.86)	0.83	(0.81,0.86)	0.89	(0.85,0.91)	0.003 (0.001)	0.006^b^
	65–79	0.69	(0.65,0.72)	0.68	(0.65,0.72)	0.72	(0.70,0.75)	0.73	(0.70,0.76)	0.75	(0.72,0.78)	0.78	(0.76,0.81)	0.78	(0.73,0.81)	0.005 (0.001)	7.6E-07^b^
	80+	0.53	(0.45,0.63)	0.65	(0.57,0.73)	0.63	(0.56,0.71)	0.60	(0.53,0.66)	0.64	(0.58,0.70)	0.68	(0.62,0.73)	0.63	(0.54,0.72)	0.007 (0.003)	0.015^b^
	Morphology																
	Non-inv pap carcinoma LG	0.85	(0.79,0.89)	0.92	(0.87,0.95)	0.91	(0.87,0.95)	0.93	(0.88,0.96)	0.91	(0.87,0.94)	0.94	(0.90,0.97)	0.93	(0.84,0.97)	0.003 (0.001)	0.001^b^
	Non-inv pap carcinoma HG	0.94	(0.18,1.00)	0.76	(0.63,0.85)	0.84	(0.71,0.92)	0.80	(0.66,0.88)	0.82	(0.72,0.88)	0.87	(0.80,0.92)	0.88	(0.77,0.94)	0.004 (0.002)	0.050
	Dysplasia	0.76	(0.56,0.87)	0.63	(0.47,0.76)	0.76	(0.61,0.86)	0.83	(0.68,0.92)	0.85	(0.67,0.93)	0.83	(0.66,0.92)	0.74	(0.54,0.86)	0.004 (0.003)	0.155
	Carcinoma in situ	0.79	(0.45,0.93)	0.62	(0.48,0.74)	0.64	(0.51,0.75)	0.64	(0.53,0.73)	0.84	(0.69,0.92)	0.83	(0.71,0.90)	0.91	(0.70,0.97)	0.013 (0.004)	1.6E-04^b^
	Inv carcinoma	0.47	(0.43,0.51)	0.49	(0.45,0.52)	0.51	(0.48,0.55)	0.50	(0.47,0.54)	0.54	(0.50,0.57)	0.55	(0.52,0.58)	0.58	(0.54,0.63)	0.003 (0.001)	2.3E-04^b^
	Stage																
	Localised	0.73	(0.69,0.76)	0.76	(0.73,0.79)	0.77	(0.74,0.79)	0.77	(0.74,0.79)	0.80	(0.78,0.82)	0.82	(0.80,0.84)	0.81	(0.77,0.84)	0.004 (0.001)	7.0E-06^b^
	Regional advanced	0.25	(0.17,0.34)	0.27	(0.20,0.36)	0.28	(0.19,0.38)	0.23	(0.15,0.31)	0.25	(0.18,0.32)	0.27	(0.21,0.33)	0.22	(0.04,0.49)	<0.001 (0.002)	0.991
	Distant advanced	0.05	(0.02,0.11)	0.08	(0.04,0.14)	0.05	(0.01,0.15)	0.04	(0.01,0.12)	0.09	(0.04,0.17)	0.07	(0.03,0.12)	0.05	(0.01,0.030)	−0.001 (0.002)	0.509
Women	Age (years)																
	0–49	0.90	(0.76,0.96)	0.82	(0.68,0.90)	0.87	(0.75,0.94)	0.95	(0.84,0.99)	0.82	(0.67,0.91)	0.79	(0.65,0.88)	0.78	(0.61,0.89)	−0.003 (0.003)	0.409
	50–64	0.78	(0.71,0.84)	0.85	(0.78,0.90)	0.79	(0.72,0.84)	0.84	(0.78,0.89)	0.82	(0.76,0.86)	0.89	(0.84,0.92)	0.86	(0.77,0.92)	0.004 (0.002)	0.032^b^
	65–79	0.61	(0.56,0.66)	0.64	(0.59,0.69)	0.65	(0.60,0.69)	0.72	(0.67,0.76)	0.74	(0.69,0.79)	0.72	(0.67,0.76)	0.70	(0.63,0.77)	0.008 (0.001)	1.5E-05^b^
	80+	0.51	(0.40,0.62)	0.59	(0.48,0.70)	0.52	(0.42,0.61)	0.53	(0.44,0.62)	0.51	(0.44,0.59)	0.59	(0.51,0.66)	0.68	(0.56,0.79)	0.004 (0.003)	0.195
	Morphology																
	Non-inv pap carcinoma LG	0.92	(0.82,0.96)	0.94	(0.83,0.98)	0.90	(0.82,0.94)	0.92	(0.85,0.96)	0.90	(0.84,0.94)	0.91	(0.85,0.95)	0.89	(0.78,0.95)	−0.001 (0.001)	0.585
	Non-inv pap carcinoma HG	0.74	(0.36,0.92)	0.90	(0.34,0.99)	0.71	(0.51,0.83)	0.88	(0.27,0.99)	0.79	(0.60,0.90)	0.81	(0.68,0.89)	0.88	((0.69,0.95)	<0.001 (0.005)	0.986
	Dysplasia	0.52	(0.35,0.66)	0.65	(0.46,0.79)	0.84	(0.46,0.96)	0.78	(0.54,0.91)	0.96	(0.11,1.00)	0.78	(0.57,0.90)	0.93	(0.46,0.99)	0.011 (0.005)	0.035^b^
	Carcinoma in situ	0.39	(0.15,0.62)	0.60	(0.41,0.75)	0.73	(0.52,0.86)	0.81	(0.57,0.93)	0.62	(0.50,0.72)	0.71	(0.51,0.84)	0.83	(0.29,0.97)	0.010 (0.006)	0.082
	Inv carcinoma	0.43	(0.38,0.48)	0.43	(0.37,0.48)	0.42	(0.37,0.47)	0.46	(0.41,0.51)	0.45	(0.40,0.50)	0.51	(0.46,0.56)	0.50	(0.43,0.57)	0.003 (0.001)	0.010^b^
	Stage																
	Localised	0.70	(0.66,0.74)	0.74	(0.69,0.78)	0.70	(0.66,0.73)	0.76	(0.72,0.79)	0.77	(0.74,0.80)	0.79	(0.76,0.82)	0.79	(0.74,0.83)	0.004 (0.001)	9.2E-06^b^
	Regional advanced	0.14	(0.06,0.24)	0.19	(0.10,0.29)	0.18	(0.10,0.28)	0.30	(0.20,0.40)	0.18	(0.11,0.26)	0.25	(0.16,0.35)	0.30	(0.18,0.43)	0.004 (0.02)	0.055
	Distant advanced	0.04	(0.01,0.15)	0.09	(0.01,0.27)	0.02	(0.01,0.10)	0.04	(0.01,0.10)	0.06	(0.01,0.16)	0.11	(0.05,0.22)	0.08	(0.01,0.41)	−0.010 (0.006)	0.136

5-year relative survival proportions (Ederer II) including confidence intervals by sex, morphology group and stage stratified by 5-year diagnosis intervals from 1981 to 2014. We applied internal age-standardization based on the latest diagnostic period 2011-14. Average annual relative survival proportion changes $\left({\widehat{\beta}}_{RS}\right)$ are reported together with standard error (SE) and *p*-value for the test of a significant survival trend.

^a^The relative survival values for the latest diagnostic period are based on a period approach

Significant *p*-values (based on the 5 % threshold) are marked with^b^

**Fig. 2:**
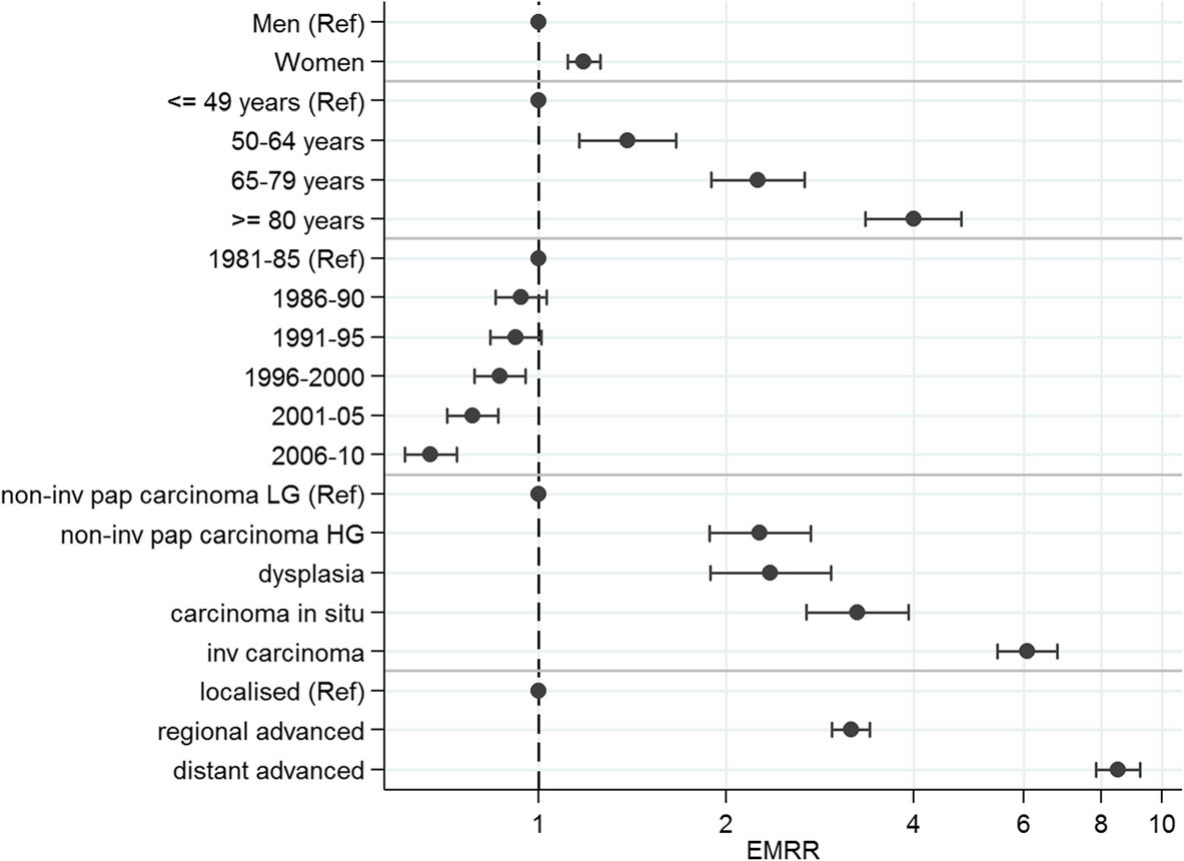
Estimated excess mortality rate ratios (EMRRs) and confidence intervals for sex, age group, diagnostic period, morphology and stage based on a proportional excess hazard model

We estimated the influence from the variables considered in our study (sex, age, diagnosis period, morphology group, stage) on the relative survival estimates and the results are illustrated in Fig. 2. The largest impact on relative survival had a diagnosis where the patient had metastases and/or any infiltration into surrounding areas. Patients with distant advanced tumors are 8.5 times more likely to die than patients with localized tumors (excess mortality rate ratio EMRR = 8.5, CI: 7.8–9.2). The corresponding EMRR for regional advanced tumors is 3.2 (CI: 3.0–3.4) when compared to localized tumors. The most severe diagnosis of having an invasive tumor lead to an EMRR of 6.1 (CI: 5.4–6.8) when comparing to the least severe diagnosis of a low-grade non-invasive papillary carcinoma. Carcinoma in situ (EMRR 3.2, CI: 2.7–3.9), dysplasia (EMRR 2.4, CI: 1.9–2.9) and high-grade papillary carcinoma (EMRR 2.3, CI: 1.9–2.7) also increased the EMRR significantly. The EMRR increased exponentially with age with an EMRR of 4.0 (CI: 3.3–4.8) for patients 80 years old (and older). The excess mortality rate was 1.2 times higher in women compared to men (CI: 1.1–1.3). All these effects were strongly significant.

## Discussion

This paper presents trends in incidence and survival for patients diagnosed with urothelial carcinoma of the urinary bladder in Norway between 1981 and 2014.

Incidence of UCB in men and women has significantly increased throughout the study period. While this trend was rather linear for women, for men an initial increase of incidence rates in the eighties was observed, followed by stable incidence rates. Interestingly, a similar pattern has been observed in lung cancer, which is also a smoking-related cancer type. A recent report [31] showed that the lung cancer incidence rates in men levelled off while incidence rates in females were still increasing in Norway. A possible explanation is that the trends for lung cancer and UCB both reflect the variation in smoking habits in the Norwegian population. While the percentage of daily male smokers between 16 and 74 years has been almost linearly decreasing from 1973 (52 %) to 2014 (13 %) in Norway, the corresponding percentage for female smokers kept stable from 1973 through 1999 (31–34 %) and followed the smoking trend in men thereafter [32]. In addition, the application of an age-period-cohort model indicated that birth cohort effects, which are tightly connected to smoking patterns in the population, could at least partly, explain the observed trends. Furthermore, the overall increase in the incidence of UCB can be explained by a significant increase in the incidence of non-invasive tumours. In comparison to our results, Abdollah et al [16] observed a linear increasing trend in age-standardized incidence of UCB from 21.0 to 25.5 per 100 000 person-years within the last three decades in US men. The same authors also reported an increased incidence for localized stages, which is in line with our observations. In South Australia, incidence increased during 1980 to 2004 for carcinoma in situ and invasive UCB combined [15] while invasive UCB stayed stable in Norway. However, we also observed an increase in high-grade non-invasive carcinoma (Tis, Ta) over time in men. Our data on sex differences in incidence are in line with other contemporary publications [33–35].

We have shown a small overall improvement in 5-year relative survival proportions over the past three decades. This improvement can particularly be seen in older patients and in those suffering from carcinoma in situ. There is no significant survival improvement for advanced tumors in either sex. This could be interpreted as a sign of a negligible effect of the increased use of cystectomy during the study period. Since this treatment was also increasingly used for elderly patients, the same explanation could also apply to the small but apparent survival improvement in elderly patients. However, the largest improvement was seen for flat non-invasive tumors in women and non-invasive tumors in men, which might reflect the introduction of immunotherapy (Bacillus Calmette-Guerin) in the 1990s in Norway. In South Australia, there was a decrease in 5-year survival proportions from 64 to 58 % observed during 1980 to 2004 for in situ and invasive UCB combined [15]. Survival trends in Switzerland for malignant UCB (non-invasive papillary and in situ carcinomas excluded) concluded with little survival improvement based on data from 1991 to 2010 [17]. A study investigating the variation of temporal trends with respect to sociodemographic and socioeconomic factors illustrated the underlying disparities associated with detection and treatment of UCB and indicated the “necessity of debate of developing a valid screening procedure for UCB” at least for particular risk groups [16].

Modelling excess mortality with respect to the variables included in our study confirmed advanced tumors, invasive tumors, flat tumors and high-grade tumors as well as older age as risk factors. Sex and diagnosis period also play a significant role, but to a lesser extent. Sex differences in survival have been widely discussed. Differences with respect to the underlying anatomy, delays in diagnosis in females, as well as variations in hormone receptors and tumor biology might play a key role [36–38].

Relative survival estimates rely on population life tables rather than using cause-specific death information. Pros and cons have been widely discussed with a general agreement on the use of relative survival to estimate net survival for population-based studies. The main concern about using relative survival is the estimation of the underlying expected survival in the population. The latter needs to be a comparable group from the general population, comparable also with respect to certain confounders like smoking. The use of a general population lifetable, and not a lifetable adjusted for smoking, may have led to an underestimate of relative survival [39–41]. In a recent comparison of methods for estimating net survival, the Ederer II method [25] seemed to be the best choice [42].

The main limitation of this study is that we were not able to distinguish between muscle-invasive (T2-4) and non-muscle invasive (T1) cancers because these tumors are condensed to the same morphology group in our cancer registry. However, we did have information about the existence of metastases with tumors classified into three stages: localized (non-invasive/invasive cancer without any metastasis), regional advanced (any infiltration into surrounding areas or regional metastases) and distant advanced (distant metastases) tumors. The localized group contains tumors without metastases but also included those with unrecognized metastases. The reason for that is a coding change in 1993 with respect to the latter group, which does not allow us to separate no-metastasis patients from those with unknown spread. Another drawback is that we had to use grading information based on the WHO 1973 system, such that our definition of low- and high grade is partly different from the WHO/ISUP 2004 system since we define low-grade tumors to include WHO grade 1 and 2 and high-grade tumors WHO grade 3.

## Conclusion

This study gives a comprehensive overview over incidence and survival changes related to a diagnosis of urothelial carcinoma of the urinary bladder in Norway from 1981 to 2014. To our knowledge, this is the only nationwide investigation of incidence and survival worldwide. The main conclusion is, that survival proportions increased only marginally. Thus, any changes in treatment and follow-up care did not lead to notable improvement with respect to survival of the patients. This conclusion gets support from several other studies looking at these trends over time [15–17]. High estimates of preventive cases together with high recurrence and progression rates of this particular cancer type, demands more research on prevention guidelines, diagnostic tools and treatment for bladder cancer.

## Abbreviations

CI: Confidence interval
EMRR: Excess mortality risk ratio
HG: High-grade
LG: Low-grade
UCB: Urothelial carcinoma of the urinary bladder
